# Protocol for a systematic review of the in vivo studies on radiofrequency (100 kHz–300 GHz) electromagnetic field exposure and cancer

**DOI:** 10.1186/s13643-022-01898-4

**Published:** 2022-02-16

**Authors:** Rosanna Pinto, Lucia Ardoino, Paola Giardullo, Paola Villani, Carmela Marino

**Affiliations:** Italian National Agency for New Technologies, Energy and Sustainable Economic Development – ENEA, Division of Health Protection Technologies, CR ENEA Casaccia- via Anguillarese 301, 00123 Santa Maria di Galeria, RM Italy

**Keywords:** Electromagnetic fields, Radiofrequency, In vivo studies, Carcinogenicity, Systematic review

## Abstract

**Background:**

An Italian project aims to review the scientific literature on the possible carcinogenicity of radiofrequency (100 kHz–300 GHz) electromagnetic field (RF-EMF) exposure. The ENEA team has to carry out a systematic review of the in vivo studies on this topic.

**Objectives:**

Development of a protocol for a systematic review (meta-analysis included) to investigate the potential carcinogenic risk following RF-EMF in vivo exposure to doses above or within legal limits. The aims of this review are (1) to provide a descriptive and, if possible, a quantitative summary of the results of the examined RF-EMF in vivo studies, together with an assessment of the consistency of observations and of the causes of heterogeneity, and (2) to assess the weight of evidence to support or refute the hypothesis of carcinogenic effects caused by RF-EMF exposure and to draw conclusions about the potential for carcinogenicity of RF-EMF exposure.

**Methods:**

We will search for relevant studies in electronic academic databases and in the reference list of selected papers and reviews on the topic, including the descriptive reviews on RF-EMF carcinogenic effect carried out by international panels of experts since 2011. The following elements of the PECO question were defined*:* experimental studies on rodents of both sexes, all ages and species, all genetic backgrounds (Population) exposed to RF-EMF alone, or in combination with other physical or chemical agents (Exposure); only studies reporting outcome data in exposed and sham control groups (Comparison); and all types of cancer with all tumor-related outcome measures (Outcome) will be included.

Only peer-reviewed articles written in English will be considered without limit in the publication date.

Eligibility criteria were defined for papers to be included. A risk of bias assessment will be performed using a tool specifically developed for animal studies. A meta-analysis will be performed, if feasible, for all outcome measures; for subgroup analysis, a minimum of 3 studies per subgroup will be required. If meta-analysis will not be possible, a narrative synthesis of the results will be reported.

**Systematic review registration:**

PROSPERO CRD42020191105

**Highlights:**

- An Italian collaborative research agreement aims to review the scientific literature on the possible carcinogenicity of RF-EMF (100 kHz – 300 GHz).
- The ENEA team will systematically review and, if possible, meta-analyse estimates the effects of in vivo exposure to RF-EMF exposure on cancer.
- The ENEA group is a multidisciplinary team of researchers with a consolidated experience both in carcinogenicity experiments and radiofrequency dosimetric assessment.
- The proposed protocol uses the NTP OHAT Approach for Systematic Review as an organizing framework.
- The proposed protocol aims to lead to the first systematic review providing a strength of evidence assessment on this topic.

**Supplementary Information:**

The online version contains supplementary material available at 10.1186/s13643-022-01898-4.

## Background

The Italian National Institute for Insurance against Accidents at Work (INAIL) and the Italian National Institute of Health (ISS) have launched the collaborative research agreement “BRiC 06/2018” Ref Y43 “*Scientific evidence on the carcinogenicity of radiofrequency electromagnetic fields*” aiming to review the scientific literature on the possible carcinogenicity of RF-EMF (100 kHz–300 GHz) provided by general population epidemiological studies, epidemiological studies on workers, in vivo animal experimental studies, and experimental studies on cells in vitro. ISS is the institution leader of the project and the Italian National Agency for New Technologies, Energy and Sustainable Economic Development (ENEA), as a partner, has to carry out a systematic review of the in vivo studies on radiofrequency RF-EMF exposure and cancer. The ENEA team is led by CM, biologist, Head of the Division Health Protection Technologies, and comprises PV and PG, biologists with a consolidate experience in experimental studies on carcinogenesis and by RP and LA, electronic engineers with a consolidate experience in dosimetry of electromagnetic fields.

In this paper, the protocol for a systematic review and, if possible, for a meta-analysis of data concerning in vivo experimental animal studies and cancer is presented.

The protocol is registered in PROSPERO with the following registration number: CRD42020191105.

## Rationale

Exposure to electromagnetic fields (EMF) has grown steadily over the past decades and in 2011 the International Agency for Research on Cancer (IARC) classified radiofrequency electromagnetic fields (RF-EMF) as “possibly carcinogenic to humans,” thus allocating them to Group 2B of its classification system [15]. The possible carcinogenic effects of RF-EMF have been investigated in laboratory animals since the early 1980s because in vivo studies have an important role in supporting the evidence derived from epidemiological studies investigating carcinogenic effects of RF exposure on the general population in daily life. After IARC monograph publication in 2013, several international panels of experts carried out descriptive literature reviews on this topic ([1–3, 6, 10, 16, 24, 25, 28, 30] - [8, 26] (see the acronyms table)). Nowadays, no systematic reviews on carcinogenic RF effects are available; this is the first attempt of a systematic review of in vivo studies on radiofrequency carcinogenic effects. RF-EMF animal studies on carcinogenesis cover a wide range of experimental situations, in terms of exposure modality, study design, and biological endpoints. This peculiarity has ambivalent effects: on one hand, it is very difficult to make a univocal classification of the studies and, consequently, to compare the results for a comprehensive analysis, on the other hand, the diversity of studies cover a wide range of experimental scenarios, providing a reasonably well insight into the effects of RF-EMF exposure on carcinogenesis in laboratory animals.

Regarding the exposure, different frequencies were used, from a few hundred MHz to some GHz, with different modulation schemes, e.g., continuous wave (CW), pulsed, Global System for Mobile Communication (GSM)-like signals, and Code Division Multiple Access (CDMA)-like signals. The used signals are mainly those of mobile communications, but the variety of signals and platforms, over the years and in different countries of the world, does not simplify the task of evaluating and synthesizing the stream of evidence for a review. In telecommunications, unlike other applications, lies the greater diversification of signals. The other signals studied were mainly at the frequency of 2.45 GHz, used for Wireless Fidelity (WiFi) system and microwave ovens. One of the main critical issues in the RF-EMF experimental in vivo studies is the dosimetry [7, 18, 23], the assessment of the effective dose induced in the RF-EMF exposed object/subject in terms of specific absorption rate (SAR, W/kg). Studies differ widely both in the RF dose of treatment, from non-thermal SAR levels to high SAR levels associated with an increase in body temperature, and in the method of exposure (i.e., full-body vs localized exposure, restrained vs free animals moving within large cages), together with not always appropriate use of control groups. Finally, the effect of RF-EMF exposure was studied both using RF-EMF alone and in synergy with other physical and chemical agents as well-known carcinogens.

In addition to the difficulty of establishing a common yardstick for evaluating such heterogeneous studies, it should be also considered that the literature experimental data on the carcinogenic effects of RF-EMF often report conflicting conclusions; even contradictory results are sometimes obtained in replicas of experiments.

## Objective

Keeping the previous considerations in mind, all study designs and all tumor-related outcome measures will be analyzed aiming:

- To revise and summarize, narratively and quantitatively (if feasible), findings from the available in vivo studies on RF-EMF (100 kHz–300 GHz) exposure and cancer;
- To assess the confidence and level of evidence in support of the carcinogenicity of RF-EMF provided by experimental studies in animals to estimate the potential carcinogenic risk following exposure to doses above or within legal limits.

## Methods

The methodology for systematic reviews for in vivo studies, described in the “Handbook for Conducting a Literature-Based Health Assessment Using OHAT Approach for Systematic Review and Evidence Integration edited by the National Toxicology Program (NTP) - Office of Health Assessment and Translation [21],” will be applied. This guide proposes new methods for evidence-based evaluation of non-human toxicological studies, including mechanistic studies, starting from those methods already in use, but less fit-for-purpose.

Other handbooks will be consulted to upgrade the methodology regarding specific aspects of experimental studies [11–13].

This protocol adheres to the preferred reporting items for systematic review and meta-analysis protocol statements (PRISMA-P) [19, 27].

### Eligibility criteria

The eligibility criteria were defined using the Population, Exposure, Comparison, Outcome (PECO) strategy [22].

### Types of populations

Only articles reporting experimental studies on rodents of both sexes, of all ages and species, of all genetic backgrounds (wild type, transgenic and tumor-prone animal models) will be included in this review.

### Types of exposures

We will include studies where experimental animals were exposed to electromagnetic fields (100 kHz–300 GHz) alone or in combination with other physical or chemical agents. The exposure system details, exposure modality, and dosimetric assessment should be reported. Studies regarding exposure to extremely low frequency (ELF), infrared, visible, and ultraviolet (UV) radiations, as well as theranostic applications, will be excluded from this review. Papers with lack of dosimetric information or with the use of inappropriate sources for the generation of the incident field will be considered not eligible.

### Types of comparators

To be eligible for inclusion, studies should report outcome data in the exposed groups and in a sham control group (a control group simulating all environmental conditions and stress factors of exposed animals, but in the absence of RF-EMF exposure).

### Types of outcomes

All types of cancer will be considered as well as all tumor-related outcome measures (e.g., incidence, tumor multiplicity, tumor volume, progression), while articles concerning genotoxicity and oxidative stress only will be excluded.

### Types of studies

Only peer-reviewed articles written in English will be considered; all publication dates will be included. Reviews will be excluded as a source of original data, but retained as a check of the bibliographic research.

### Information sources and search strategy

#### Electronic academic databases

The following databases will be used for the search:

PubMed: www.pubmed.ncbi.nlm.nih.gov;

EMF-Portal: www.emf-portal.org/en.

Only English language peer-reviewed papers, without restrictions on the year of publication, will be included in the review.

The search strategy (query, see supplementary material) will be composed of seven distinct elements, separated by appropriate logical operators:

1. RF-EMF (identifying exposure);
2. In vivo studies, animal studies, rodents, mice, rats (identifying population);
3. Carcinogenicity, cancer/tumor/neoplasia, neoplastic and non-neoplastic lesions, tumor induction/co-promotion (identifying outcome);
4. Body organs and tissues (identifying biological target);
5. Elements intended to exclude emissions different from RF-EMF: static and ELF electric and/or magnetic fields, UV radiations alone, infrared and visible radiation, ionizing radiation (identifying exclusion criteria for exposure);
6. Elements intended to exclude theranostic applications: RF and microwave (MW) ablation, hyperthermia and MW imaging (identifying exclusion criteria for study type);
7. Elements intended to exclude other studies (i.e. observational).

#### Citation searching

The reference lists of the selected papers and reviews on the topic, including descriptive reviews carried out by international panels of experts since 2011, will be screened to find potentially relevant papers that may have escaped the first search (*Electronic academic databases*).

The NTP-OHAT Handbook (March 2019) specifies that it is possible to identify relevant publications that are not commercially published or are not readily available to the public yet. These publications (e.g., technical reports from government agencies or scientific research groups, working papers from research groups or committees) may include or summarize unpublished data.

All references coming from these last sources will be marked as “provided from other sources” in the study selection flow diagram.

### Selection process

All potentially relevant articles will be screened for eligibility in two stages: a first stage in which the articles will be selected, on the basis of title and abstract, by three authors (RP, PV, PG), a second stage, in which the full text of the remaining papers will be independently reviewed by two groups of investigators, each composed by one biologist and one expert in EMF dosimetry (RP, PG group 1 and LA, PV group 2). Disagreements and technical uncertainties will be discussed and resolved between review authors.

The exclusion criteria will be prioritized according to the following list:

1. Not an original full research paper (e.g., reviews, editorials, letters);
2. Not English paper;
3. Not animal (rodents) studies;
4. Not cancer endpoints;
5. Exposure outside the 100 kHz–300 GHz range;
6. Lack of dosimetric information;
7. No sham/control group;
8. Theranostic application.

### Data collection process

#### Data extraction

The data extraction form will be defined and agreed upon before the start of paper analysis. The eligible papers will be equally divided between group 1 and group 2 to independently extract numerical data from text, tables, or figures of each article. In case of missing outcome data, if possible, the review team will contact the authors at least once by mail.

#### Data items

The extracted data will include:

- Study design (number of experimental groups, control group(s), number of animals per group, randomization and blinding),
- Study type (e.g., cancer induction, co-carcinogenesis studies),
- Animal model (species, strain, sex, genotype of animals (wild type, transgenic)),
- Timing of treatment (i.e., hours per day, days per week and total period; age or life stage at the start of exposure),
- Exposure details (frequency, modulation, dose and type of exposure (whole body vs localized exposure, restrained vs freely moving animals), exposure system),
- Primary outcome(s): all tumor-related outcome measures (incidence, tumor multiplicity, tumor volume, progression, latency, survival),
- Secondary outcome(s): all parameters related to animal health conditions evaluated at the end of life/experiment,
- Method to assess the endpoints,
- Data analysis and statistical evaluation,
- Authors, year of publication, title, journal.

We will also extract data on potential conflict of interest in included studies.

### Risk of bias assessment

The internal validity and the quality of eligible studies will be evaluated using the approach recommended by NTP-OHAT [21] for animal studies. It provides detailed instructions for assessing how potential sources of distortion may have affected the reliability of the results.

The nine included risk of bias criteria are:

1. Randomized exposure level;
2. Allocation concealment of study groups;
3. Evaluation in the study design or analysis of possible important confounding and modifying variables;
4. Blinding of research personnel;
5. Confidence in the exposure characterization (dosimetry);
6. Confidence in the outcome assessment;
7. All measured outcomes reported;
8. Attrition/exclusion rate;
9. Possible conflicts of interest.

Regarding item 3, possible important confounding factors are, for example, uncontrolled temperature increases in the exposed animals or any other difference in the experimental conditions between exposed and comparison groups. Since the presence of sham control group(s) is mandatory for the inclusion of the study in the systematic review, it is useless to remark the importance of identical exposure conditions as reported in NTP-OHAT [21]. Two groups of authors (RP, PG and LA, PV) will independently assess these criteria at the individual study level and they will classify the studies according to the following ratings: “++” definitely low risk of bias, “+” probably low risk of bias, “−” probably high risk of bias, or “−−“ definitely high risk of bias. Disagreements in the assessment will be discussed between the authors and resolved by consensus. Furthermore, using the OHAT approach, individual studies will be placed into three quality categories based on the risk-of-bias ratings. The key criteria for determining the highest weight in the quality of the study are as follows: (1) confidence in the exposure characterization, (2) confidence in the outcome assessment, and (3) evaluation in the study design or analysis of possible important confounding and modifying variables. The remaining criteria will be given less weight in determining the quality of the study.

### Data synthesis criteria and strategy

Structured descriptive summary of eligible studies will include the following items:

1. Experimental design (e.g., induction/promotion, co-promotion);
2. The animal model used;
3. Age of animals at the start of treatment;
4. The developmental stage of animals at treatment and outcome assessment (e.g., mating and/or pregnancy status);
5. Exposure type and level;
6. Outcome(s) reported;
7. Type of data (e.g., continuous or discrete), statistics presented in the paper, possibility to access raw data;
8. Variation in the degree of risk of bias at the individual study level.

A meta-analysis, if feasible, will proceed according to the following main sequence [29]:

1. Calculating an effect size measure for each comparison, according to the typology of data (effect size) of the considered studies,
2. Weighing the effect size measure according to the effect model adopted (random or fixed),
3. Calculating the summary effect size,
4. Calculating the heterogeneity and the extent to which the predefined study design characteristics explain this heterogeneity,
5. Evaluating the subgroups analysis;
6. Checking the presence of meta-bias (e.g., attrition bias, publication/duplicate bias, detection bias).

#### Effect size measures

When studies have used the same form of intervention and comparator (sham), with the same outcome measure we will pool the results in the appropriate effect model, using standardized mean difference or response ratio for continuous data (outcome) and risk ratio or risk difference for dichotomous data (outcome).

If necessary, measures of absolute effect will be converted into relative effect measures, to ensure comparability of effect estimate and facilitate the meta-analysis that will be performed for all outcome measures.

A minimum of 3 studies per group/subgroup will be required [12, 13, 20].

#### Effect size models

Because of the exploratory nature of animal studies, random effect model will be used as a first choice. Fixed-effect models will be used for groups of data without significant heterogeneity leading to a τ value of zero. Extracted data will be analyzed using statistical software with meta-analysis tools.

Forest plots will be constructed for each outcome, and the summary effect, standard error, and *p*-values will be determined with 95% of confidence interval [5].

#### Heterogeneity

Heterogeneity among studies will be assessed with Cochran *Q* test and further quantified by *I*^2^ statistics. If studies will be affected by high (*I*^2^ = 75% or greater) heterogeneity, a narrative synthesis will be done.

#### Additional analysis

Heterogeneity in the results of the meta-analysis may be observed. Variations in the effect size across sub-sets of studies are expected, for instance, if there are differences between species or between exposure levels and exposure modalities. Thus, subgroup analysis or meta-regression may give insight into the relation between study characteristics and the effect size, to find an explanation.

The regression analysis will be performed for different exposure levels (SAR) and for different exposure times.

If meta-analysis is not possible, data will be reported through the descriptive summary.

### Quality of evidence assessment

We will apply the confidence rating approach based primarily on guidance from the Grading of Recommendations Assessment, Development and Evaluation (GRADE) Working Group [4, 9]. The GRADE framework is often applied to evaluate the quality of evidence and the strength of recommendations for outcomes reported in systematic reviews [11] on human and animal studies. We will assess the quality of evidence for the entire body of evidence by each outcome, with any disagreements resolved by an independent review author. We will downgrade the quality of evidence for the following five GRADE reasons: (i) risk of bias, (ii) inconsistency, (iii) indirectness, (iv) imprecision, and (v) publication bias.

We will grade the evidence, according to the three Navigation Guide standard quality of evidence ratings: “high,” “moderate,” and “low.” Within each of the relevant domains, we will rate the concern for the quality of evidence, using the ratings “none,” “serious,” and “very serious.” We will start at “high” for randomized studies: quality will be downgraded for no concern by nil grades (0), for a serious concern by one grade (− 1), and a very serious concern by two grades (− 2). We will upgrade the quality of evidence for the following other reasons: large effect, dose-response and plausible residual confounding and bias.

### Strength of evidence assessment

We will apply a modified version of the Navigation Guide methods [31] for the nonhuman evidence [17]. The rating will be based on a combination of four criteria: (I) sufficient evidence of toxicity, (II) limited evidence of toxicity, (III) inadequate evidence of toxicity, or (IV) evidence of lack of toxicity, which in turn are based on the criteria used by the International Agency for Research on Cancer [14].

## Discussion

This systematic review of in vivo studies of carcinogenesis from exposure to RF-EMF will respond to the need for clarity emerged in the scientific community following the publication of several studies with contradictory results. In our opinion, this systematic review will contribute to assess the extent of the epidemiological evidence on this topic, in a reproducible and rigorous way.

We are not aware of published systematic reviews and meta-analyses addressing this issue. The World Health Organization’s (WHO) Radiation Program has an ongoing project to assess potential health effects, included carcinogenesis, of exposure to radiofrequency electromagnetic fields, in the general and working population, throughout systematic reviews.

Any changes made to this protocol will be reported in PROSPERO and in the final manuscript.

The results of this systematic review will complement the other results of the main project “BRiC 06/2018” Ref Y43 “Scientific evidence on the carcinogenicity of radiofrequency electromagnetic fields” on epidemiological and experimental in vitro studies, with the common aim to carry out a systematic review of scientific evidence on the carcinogenicity of RF-EMF (100 kHz–300 GHz) exposure by methods useful to minimize biases in the identification-selection-synthesis of studies and in the formulation of conclusions.

These results and meta-analysis will be presented at conferences and published in a peer-reviewed journal. Moreover, a final public event was scheduled to present the overall results of the project.

## Supplementary Information

**Additional file 1.**

## Data Availability

The authors have the access to all scientific databases, in order to collect all relevant papers for the systematic review.

## Abbreviations

AGNIR: Advisory Group on Non-Ionising Radiation (UK)
ANSES: Agence Nationale de Sécurité Sanitaire de l’Alimentation de l’Environnement et du Travail (France)
ARPANSA: Australian Radiation Protection and Nuclear Safety Agency
CCARS: Comité Científico Asesor en Radiofrecuencias y Salud (Spain)
ELF: Extremely low frequency
ENEA: Italian National Agency for New Technologies, Energy and Sustainable Economic Development
FDA: Food and Drug Administration
GRADE: The Grading of Recommendations Assessment, Development and Evaluation
HCN: Health Council of the Netherlands
IARC: International Agency for Research on Cancer
ICHENF: Interagency Committee on the Health Effects of Non-Ionizing Fields (New Zealand)
ICNIRP: International Commission for Non-Ionizing Radiation Protection
INAIL: National Institute for Insurance against Accidents at Work
ISS: Italian National Institute of Health
MW: Microwave
NTP: National Toxicology Program
OHAT: Office of Health Assessment and Translation
RF-EMF: Radiofrequency electromagnetic field
RSC-EC: Royal Society of Canada Expert Committee
SAR: Specific absorption rate
SSM-SC: Scientific Council on Electromagnetic Fields (Sweden)
SCENIHR: Scientific Committee on Emerging and Newly Identified Health Risks
SSM: Swedish Radiation Safety Authority
WHO: World Health Organization
