# Supplementary material for: Protocol for a systematic review of the in vivo studies on radiofrequency (100 kHz–300 GHz) electromagnetic field exposure and cancer
Source: Syst Rev. 2022 Feb 16;11:29. doi: 10.1186/s13643-022-01898-4 (PMC8848792; doi:10.1186/s13643-022-01898-4)
Supplement: Supplementary file 1 — Additional file 1. [file 13643_2022_1898_MOESM1_ESM.docx]

**Queries**

**PubMed Search**

|  | Exposure | Microwaves / adverse effects* [MH] OR electromagnetic fields [MH] OR microwave*[TIAB] OR radio waves [TIAB] OR “non-ionizing radiation” [TIAB] OR radiofrequency*[TIAB] OR mobile phone*[MH] OR cell phone* [MH] OR GSM [TIAB] OR UMTS [TIAB] OR LTE [TIAB] OR "mobile communication*" [TIAB] OR "millimeter wave*" [TIAB] |
| --- | --- | --- |
| AND | Population | Animals [MH] OR “in vivo” [TIAB] OR “animal studies” [TIAB] OR "animal model" [TIAB] OR rat*[TIAB] OR mice [TIAB] OR mouse [TIAB] OR murine [TIAB] |
| AND | Outcome | carcinogens* [MH] OR cocarcinogenesis* [MH] OR cancer [TIAB] OR carcinogenicity [TIAB] OR tumor [TIAB] OR tumorigenesis [TIAB] OR “tumor induction”[TIAB] OR “tumor promotion” [TIAB] OR “tumor regression” [TIAB] OR “tumor growth” [TIAB] OR “solid tumor” [TIAB] OR lymphoma [TIAB] OR leukemia [TIAB] OR neoplasia [TIAB] OR neoplastic [TIAB] |
| AND | Outcome | skin [TIAB] OR brain [TIAB] OR liver [TIAB] OR kidney [TIAB] OR breast[TIAB] OR bone [TIAB] OR lung [TIAB] OR acoustic nerve [TIAB] OR lymphoma [TIAB] OR leukemia [TIAB] OR Central Nervous System Neoplasms [MH] OR Brain Neoplasms / pathology* |
| NOT | Exposure | Ultraviolet Rays* [MH] OR UVA [TIAB] OR UVB [TIAB] OR “Extremely Low Frequency” [TIAB] OR ELF [TIAB] OR “50 Hz” [TIAB] OR “60 Hz” [TIAB] OR “static field” [TIAB] OR Laser [TIAB] OR acoustic [TIAB] OR ultrasound [TIAB] |
| NOT | Outcome | Ablation [TIAB] OR "Pulsed Radiofrequency Treatment" [MH] OR "Radiofrequency Therapy" [MH] OR therapy [TIAB] OR therapeutic [TIAB] OR Hyperthermia [TIAB] OR "bone transplantation*" [MH] |
| NOT | Outcome | genotoxicity [TIAB] OR genotoxic effects* [TIAB] OR "in vitro" [MH] OR "Epidemiologic Studies"[MH] OR "epidemiology"[MH] OR "diagnostic imaging" [MH] OR "Meta-Analysis"[PT] OR “Case Report”[PT] OR “Humans” [MH] OR retrospective-stud*[MH] OR prospective-stud*[MH] OR longitudinal-stud*[MH] OR follow-up stud*[MH] OR ecological-study [TIAB] OR ecological-studies [TIAB] OR Cross-Sectional Stud*[TIAB] OR Correlation-stud*[TIAB] OR cohort*[MH] OR case-control*[MH] OR cancer-registr*[TIAB] OR case-series[TIAB] OR case-referent [TIAB] OR record-link*[TIAB] OR meta-analysis[TIAB] OR case-report[PT] OR meta-analysis[TIAB] OR meta-analysis[PT] OR review [PT] |

Text for Query Box

(((((((Microwaves / adverse effects* [MH] OR electromagnetic fields [MH] OR microwave*[TIAB] OR radio waves [TIAB] OR “non-ionizing radiation” [TIAB] OR radiofrequency*[TIAB] OR mobile phone*[MH] OR cell phone* [MH] OR GSM [TIAB] OR UMTS [TIAB] OR LTE [TIAB] OR "mobile communication*" [TIAB] OR "millimeter wave*" [TIAB]) AND (Animals [MH] OR “in vivo” [TIAB] OR “animal studies” [TIAB] OR "animal model" [TIAB] OR rat*[TIAB] OR mice [TIAB] OR mouse [TIAB] OR murine [TIAB])) AND (carcinogens* [MH] OR co-carcinogenesis* [MH] OR cancer [TIAB] OR carcinogenicity [TIAB] OR tumor [TIAB] OR tumorigenesis [TIAB] OR “tumor induction”[TIAB] OR “tumor promotion” [TIAB] OR “tumor regression” [TIAB] OR “tumor growth” [TIAB] OR “solid tumor” [TIAB] OR lymphoma [TIAB] OR leukemia [TIAB] OR neoplasia [TIAB] OR neoplastic [TIAB])) AND (skin [TIAB] OR brain [TIAB] OR liver [TIAB] OR kidney [TIAB] OR breast[TIAB] OR bone [TIAB] OR lung [TIAB] OR acoustic nerve [TIAB] OR lymphoma [TIAB] OR leukemia [TIAB] OR Central Nervous System Neoplasms [MH] OR Brain Neoplasms / pathology*)) NOT (Ultraviolet Rays* [MH] OR UVA [TIAB] OR UVB [TIAB] OR “Extremely Low Frequency” [TIAB] OR ELF [TIAB] OR “50 Hz” [TIAB] OR “60 Hz” [TIAB] OR “static field” [TIAB] OR Laser [TIAB] OR acoustic [TIAB] OR ultrasound [TIAB])) NOT (Ablation [TIAB] OR "Pulsed Radiofrequency Treatment" [MH] OR "Radiofrequency Therapy" [MH] OR therapy [TIAB] OR therapeutic [TIAB] OR Hyperthermia [TIAB] OR "bone transplantation*" [MH])) NOT (genotoxicity [TIAB] OR genotoxic effects* [TIAB] OR "in vitro" [MH] OR "Epidemiological Studies"[MH] OR "epidemiology"[MH] OR "diagnostic imaging" [MH] OR "Meta-Analysis"[PT] OR “Case Report”[PT] OR “Humans” [MH] OR retrospective-stud*[MH] OR prospective-stud*[MH] OR longitudinal-stud*[MH] OR follow-up stud*[MH] OR ecological-study [TIAB] OR ecological-studies [TIAB] OR Cross-Sectional Stud*[TIAB] OR Correlation-stud*[TIAB] OR cohort*[MH] OR case-control*[MH] OR cancer-registr*[TIAB] OR case-series[TIAB] OR case-referent [TIAB] OR record-link*[TIAB] OR meta-analysis[TIAB] OR case-report[PT] OR meta-analysis[TIAB] OR meta-analysis[PT] OR review [PT])

The Query provides 112 results.

**EMF Portal Search**

From the EMF Portal Home Page the following steps were taken:

Select "Literature search";

After selecting ***only*** the following research areas

**Topics**: Experimental studies

**Frequency Ranges**: Radio Frequency (>10 MHz) + Mobile Communication

**Time Span**: complete time span

in the field Keywords, type the text "animal AND cancer".

The sequence provides 114 results.

All the results were charged on EndNote X9 to proceed to the further screening as described in the Flow Diagram.
